# The Complete Mitochondrial Genome Sequence of the Planthopper, *Sivaloka damnosus*

**DOI:** 10.1673/031.010.7601

**Published:** 2010-06-25

**Authors:** Nan Song, Ai-Ping Liang, Chuan Ma

**Affiliations:** Key Laboratory of Zoological Systematics and Evolution, Institute of Zoology, Chinese Academy of Sciences, I Beichen West Road, Chaoyang District, Beijing 100101, P.R. China

**Keywords:** A+T-rich region, Issidae, Hemiptera, phylogeny

## Abstract

The complete mitochondrial genome (mitogenome) sequence was determined from the plant hopper, *Sivaloka damnosus* Chow and Lu (Hemiptera: Issidae), a representative of the insect family Issidae. The genome is a circular molecule of 15,287 bp with a total A+T content of 76.5%. The gene content, order, and structure are identical to that in *Drosophila melanogaster*, which is considered ancestral for insects. All 13 protein-coding genes of the *S. damnosus* mitogenome have a putative inframe ATR methionine or ATT isoleucine codons as start signals. The usual termination codons (TAA and TAG) were found in 11 protein-coding genes. However, *atp6*, and *nad4* have incomplete termination codons. All tRNAs show stable canonical clover-leaf structures similar to other insect mitochondrial tRNAs, except for tRNA^*Ser*(*AGN*)^, which has a reduced DHU arm. The A+T-rich region or putative control region includes two extensive repeat regions. The first repeat region is composed of two sets of complicated repeat units, and these repetitive sequences are arranged alternately; the second contains ten 20 bp tandemly repetitive sequences. In the phylogenetic analyses based on protein-coding genes, Cicadomorpha is a sister to Fulgoromorpha+Sternorrhyncha, and Heteroptera is a sister to all other Hemiptera.

## Introduction

During the last decade, the number of the mitogenomes, i.e. mitochondrial genomes, of arthropods has increased, as a result of the development of genomic technologies, the interest in mitogenome organization and evolution (Boore et al. 2005), and the use of complete mitogenomes in phylogenetic inferences (Carapelli et al. 2007). In general, the insect mitogenome is a circular, double-stranded molecule of 14–19 kb in length that comprises a set of 37 genes for 22 tRNAs, 2 rRNAs, and 13 proteins. Additionally, the insect mitogenome contains a control region known in insect mitochondrial DNA (mtDNA) as the A+T-rich region, which contains signals for the transcription of both strands in *Drosophila* species (Clary and Wolstenholme 1985), the replication of one of the two strands (Clary and Wolstenholme 1987), and source of length variation in the mitogenome (Inohira et al. 1997).

The order Hemiptera is the largest nonholometabolan insect assemblage. Three suborders are recognized within the Hemiptera: the Heteroptera (true bugs), Stremorrhyncha (aphids, scale bugs, whiteflies, and psyllids) and the Auchenorrhyncha (planthoppers, leafhoppers, spittlebugs, and cicadas) (Carver et al. 1991). The interrelationships of these three suborders have traditionally been controversial, particularly the phylogenetic position of Fulgoromorpha (Hemiptera: Fulgoroidea). More mitogenome sequences might help to resolve the phylogenetic relationships of hemipteran insects. Complete or nearly complete mtDNA sequences of Hemiptera are available in sequence databases including the spittlebug, (Stewart and Beckenbach 2005), psyllid (Thao et al. 2004), leafhopper (GenBank accession No. NC_006899, Baumann and Baumann), planthopper (Song and Liang 2009), 2 aphids (Thao et al. 2004; GenBank accession No. NC_011594, Moran et al.), 6 whiteflies (Thao et al. 2004) and 16 true bugs (Dotson and Beard 2001; Hua et al. 2008).

The Family Issidae, belonging to Fulgoroidea, is composed of more than 1,200 species worldwide. Morphological (Emeljanov 1999) and molecular (Bourgoin et al. 1997; Yeh et al. 1998, 2005; Urban and Cryan 2007) evidence indicates that the Issidae is not a natural group but are paraphyletic relative to other planthoppers. *Sivaloka damnosus* Chow and Lu (Hemiptera: Fulgoroidea: Issidae) is one of the most common and widely distributed issids in the north of China, and is a serious pest of forests. Fragments of the mitochondrial genes encoding *16S rRNA* and *Cyt b* (Yeh et al. 1998; Yeh et al. 2005) of some issid species have already been sequenced and utilized in phylogenetic studies. However, the genetic sequence of the complete mtDNA of any issid is not yet available. A better understanding of the phylogenetic relationships in Hemiptera and the phylogenetic position of Issidae requires an expansion of taxon and mitogenome samplings. In this paper, we report the complete mtDNA sequence of *S. damnosus* and its annotated results.

## Materials and Methods

### Sample and DNA extraction

An adult of *S. damnosus* was collected in Zhejiang Province, China. The specimen was morphologically identified and preserved in 100% ethanol and stored at -80° C in the Key Laboratory of Zoological Systematics and Evolution, Institute of Zoology, Chinese Academy of Sciences.

The mitochondria isolation for the species was performed according to Tamura and Aotsuka (1988), with some modifications. The muscle tissue under pronotum was homogenized in 2 ml of chilled buffer (220 mM mannitol, 70 mM sucrose, 5 mM Tris, 2 mM EDTA, pH 8.0), and centrifuged at 800 g for 10 min at 4° C to pellet the nuclei and cellular debris. After the resultant supernatant was recovered by centrifugation at 3600×*g* for 10 min at 4° C, 1 ml homogenizing mixture was added to the precipitate and centrifuged at 12,000 g for 10 min at 4°C to pellet the mitochondria.

A modified method of the salt-extraction protocol was used to extract mtDNA from the isolated mitochodria (Aljanabi and Martinez 1997). The pellet was digested in the protease buffer (100 mM Tris, 40 mM NaCl, 2 mM EDTA, 10% SDS, 20 mg/ml proteinase K) at 55° C for 2–3 hr. The solution was mixed with 250 µl 5.3 M NaCl, and centrifuged at 1400 g for 10 min at 4° C. After 560 µl isopropanol was added to the supernatant, the mixture was chilled at -20° C for 30 min and pelleted by centrifugation at 12,000 g for 15 min at 4°C. The pellets were washed with 75 % ethanol and allowed to air dry. DNA was dissolved in 100 µl of ddH2O and one-tenth dilutions were used as template in PCR.

### PCR amplification, cloning and sequencing

**Figure 1.**
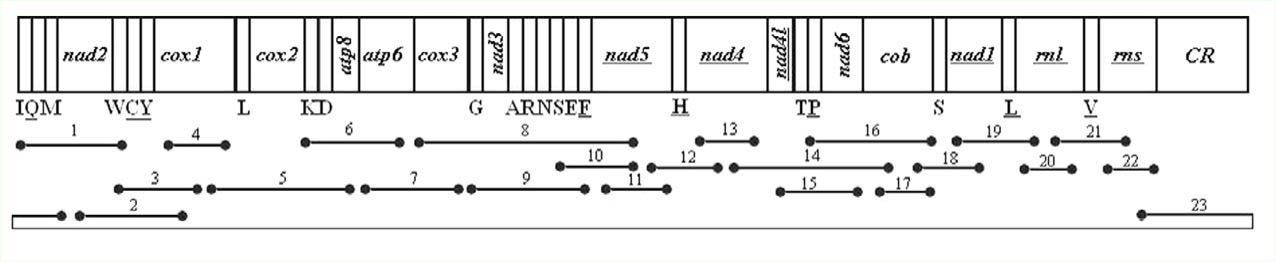
Schematic representation of amplification strategy employed for the *Sivaloka damnosus* mitochondrial genome. Lines below the linearized genome map represent the amplification products. High quality figures are available online.

The mitogenome was amplified in overlapping PCR fragments (Figure 1 shows a schematic map of the amplification fragments, and primers are shown in Table 1). Initial amplifications were conducted using sets of heterologous primers that we have developed based on aligned insect mitochondrial sequences. Then, based on the obtained sequence, specific primers were designed to amplify the rest of the mitogenome. Large fragments were obtained using the QIAGEN Long Taq DNA polymerase (Qiagen, www.qiagen.com) under the following conditions: 2 min at 96° C, followed by 30 cycles of 10 s at 98° C, and 10 min at 68° C. The final elongation was continued for 10 min at 72° C. For the small fragments, Qiagen Taq DNA polymerase was used in PCR reaction under the following conditions: 5 min at 94° C, followed by 30 cycles of 50 s at 94° C, 50 s at 50° C, and 1–2 min at 72° C. The final elongation step was continued for 10 min at 72° C. These PCR products were analyzed by 1.0% agarose gel electrophoresis.

PCR products of 1200 bp (fragment 1–7, 9– 13, 15–22 and 24 in Figure 1) were directly sequenced after purification, whereas the PCR products of 1.2–2.5 kb (fragment 8, 14 and 23 in Figure 1) were cloned into pBS-T Easy vector (Qiagen) and the resultant plasmid DNA was isolated using the TIANprp Midi Plasmid Kit Purification System (Qiagen). For each larger PCR product, at least two independent clones were sequenced to ensure that we obtained the consistent sequence. DNA sequencing was performed using the BigDye Terminator Cycle Sequencing Kit and the ABI 373 OXL Genetic Analyzer (Applied Biosystems, www.appliedbiosystems.com). All fragments were sequenced from both strands.

Sequence assembly, annotation, and analysis Sequences alignment and nucleotide composition calculations were conducted with MEGA 4 (Tamura et al. 2007). The sequences on the minority strand were reversely complemented in EditSeq (DNAStar, www.dnastar.com), and the neighboring sequences were aligned by ClustalW (Thompson et al. 1994) version 1.6 as implemented in MEGA 4 to find the overlapping regions. With the help of MEGA4 and EditSeq, the sequence assembly and annotation were conducted in the Staden sequence analysis package (Staden et al. 2000). The locations of protein-coding genes and rRNA genes were identified by comparison with those of other insects, while tRNA genes were identified using the tRNAscan-SE server (Lowe and Eddy 1997). Potential secondary structure folds in the A+T-rich region were predicted using Mfold v. 3.2 (Zuker 2003, http://www.bioinfo.rpi.edu/applications/mfold). Sequence data are available from NCBI (http://www.ncbi.nlm.nih.gov/) under accession number: FJ360694.

**Table 1.**
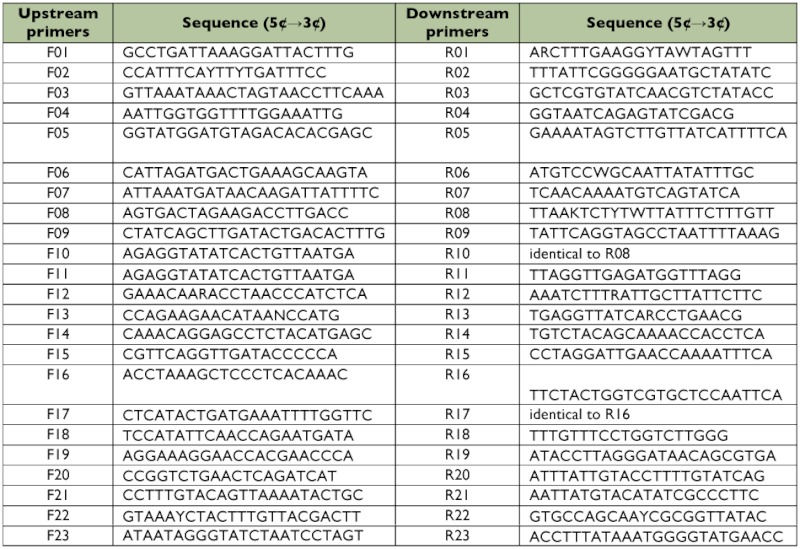
Primers used in sequencing *Sivaloka damnosus* mtDNA.

### Phylogenetic analyses

Thirty-one complete or nearly complete mitogenomes were included in the analyses (Table 2). One species from Orthoptera and one species from Psocoptera were selected as outgroups. The nucleotide sequences of 13 protein-coding genes and 2 rRNA genes were used to reconstruct the phylogenetic relationships in Hemiptera. Each gene was individually aligned using ClustalW, and 13 protein-coding genes and 2 rRNA genes were respectively concatenated. In order to test the effect of mutational saturation on the phylogenetic analyses, two kinds of protein-coding gene data sets were created: (1) DNA alignment with all three codon positions, and (2) DNA alignment including only the first and second codon positions.

The Bayesian Inference method was employed to analyze the three data sets. Bayesian analyses were conducted with MrBayes version 3.1.2 (Huelsenbeck and Ronquist 2001) with the following options: four independent Markov chains, three million generations, tree sampling every 100 generations, and the first 25% discarded as burn-in. Stationarity was considered to be reached when the average standard deviation of split frequencies was less than 0.01. Bayesian posterior probabilities (BPP) were estimated on a 50% majority rule consensus tree of the remaining trees.

**Table 2.**
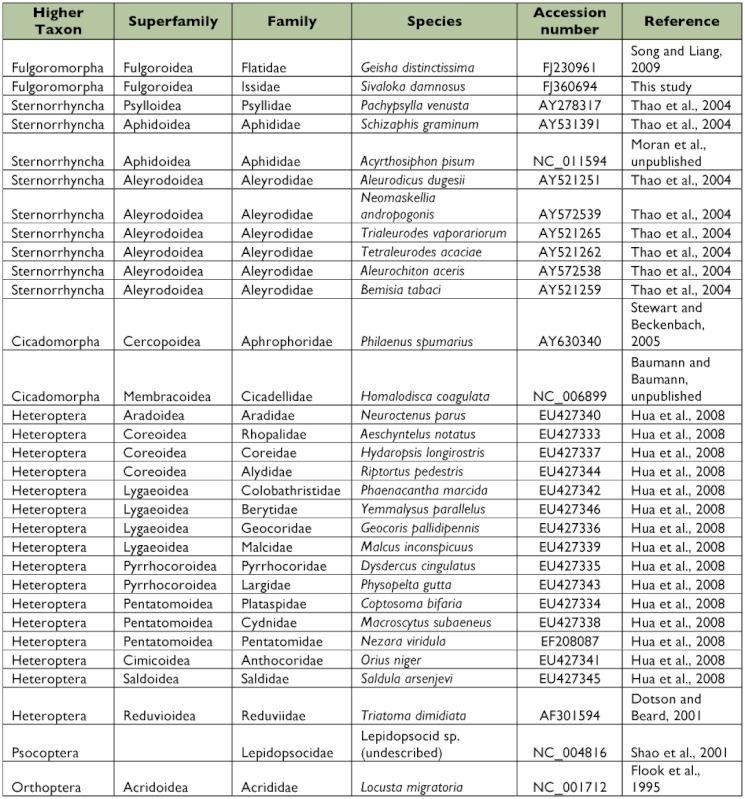
List of taxa used in the phylogenetic analysis.

## Results

### Genome structure, organization, and composition

The complete mitogenome of *S. damnosus* is circular, 15,287 bp in length. It has 13 protein-coding genes, 22 tRNA genes, and 2 rRNA genes (Table 3), as is also the case in other insects (Boore 1999). Only one long unassigned region is present between *srRNA* and tRNA^*Ile*^, and it is homologous to the A+T-rich region by positional homology, general structure and base composition. As with previously published insect mitochondrial sequences, the *S. damnosus* mitogenome sequence is A + T rich, at 76.5%. Nucleotide composition for features within the mitogenome are listed in Table 4.

**Table 3.**
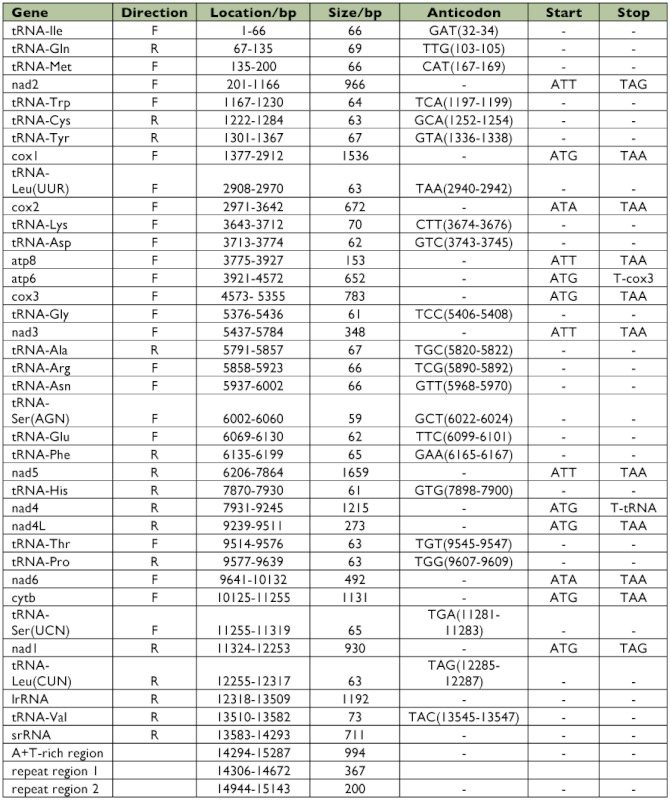
Summary of the mitochondrial genes *of Sivaloka damnosus*.

The *S. damnosus* mitochondrial genes overlap 45 bp at nine locations, varying from 1 to 9 bp with the largest one located between tRNA*^Trp^* and tRNA*^Cys^*. In other insects, the total size of overlapping regions range from 20 bp in *Bombyx mori* (Yukuhiro et al. 2002) to 152 bp in *Anopheles quadrimaculatus* (Mitchell et al. 1993). In the mitogenome of *S. damnosus*, a total of 91 bp intergenic spacer sequence is spread over in 12 regions ranging in size from 1 to 20 bp. The two largest intergenic spacers consist of 16 and 20 bases, and are located between tRNA*^Cys^* and tRNA*^Tyr^*, and between *cox3* and tRNA*^^Gly^^*, respectively.

### Protein-coding genes

Thirteen protein-coding genes were identified by comparison with other insect mitochondrial protein-coding genes found in the GenBank (BLAST searches), or by comparison with protein sequence alignments produced from the two previously published hemipteran mitogenomes: *Triatoma dimidiata* (Dotson and Beard 2001) and *Philaenus spumarius* (Stewart and Beckenbach 2005). The location and putative start and termination codons of the protein-coding genes are shown in Table 3.

**Table 4.**
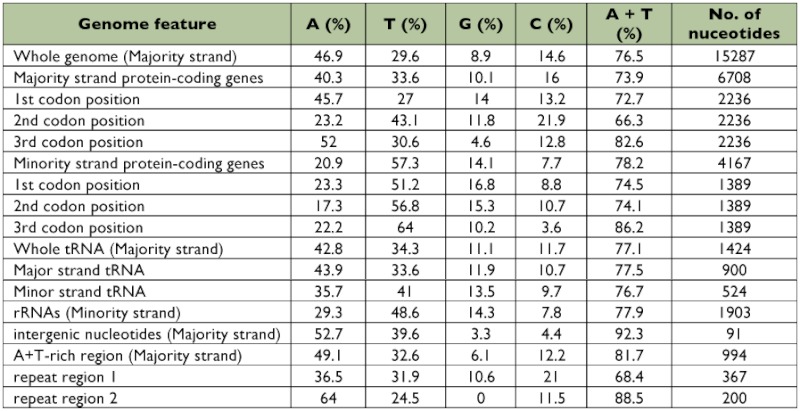
Nucleotide composition for features in the mitogenome of *Sivaloka damnosus*.

All thirteen protein-coding genes are observed to have a putative, inframe ATR methionine or ATT isoleucine codons as start signals. Seven protein-coding genes start with ATG (*cox1, atp6, cox3, nad4, nad4l, cytb*, and *nad1*), four with ATT (*nad1, atp8, nad3*, and *nad5*), and two with ATA (*cox2* and *nad6*). The start codon for *cox1* is highly variable across insects, and frequently uses noncanonical start codons (Bae et al. 2004; Kim et al. 2006). However, the *S. damnosus cox1* gene starts with the typical trinucleotide ATG.

Eleven of the 13 protein-coding genes have complete termination codons, either TAA (nine genes) or TAG (*nad1* and *nad2*), and the remaining two genes have incomplete termination codons T (*atp6* and *nad4*) (Table 3). The presence of incomplete stop codons is a common phenomenon found in a number of invertebrate mitogenomes (Crozier and Crozier 1993), and in some mammalian mitogenomes ((Bibb et al. 1981). A common interpretation for this phenomenon is that the complete termination codon is created by polyadenylation of mRNA (Ojala et al. 1981).

The relative synonymous codon usage (RSCU) values of *S. damnosus* are summarized in Table 5. A common feature of most arthropod mitogenomes sequenced to date is that the bias toward the nucleotides A and T also leads to a bias in amino acid usage. This is reflected in the proportions of amino acids with A or T versus C or G at the second and third codon positions. In Table 5, the results show a distinct preference for the use of the A or T nucleotides in the third codon positions for the twofold degenerate amino acids. At the third codon positions of fourfold degenerate amino acids, the majority-strand encoded protein genes show a preponderance of A nucleotides, whereas the protein-coding genes on the minority strand prefer T.

**Table 5.**
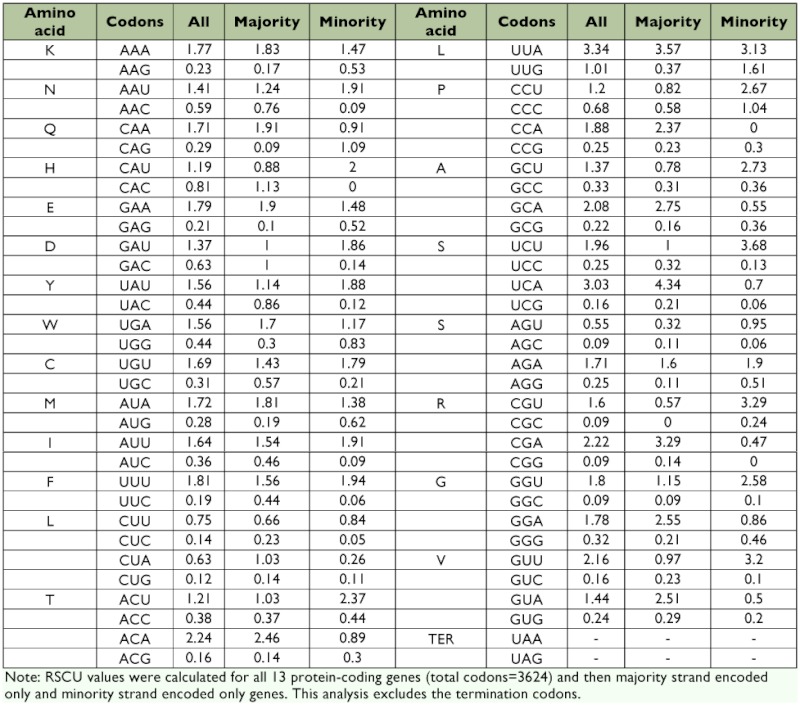
Relative synonymous codon usage for *Sivaloka damnosus*.

### Transfer RNA and ribosomal RNA genes

**Figure 2.**
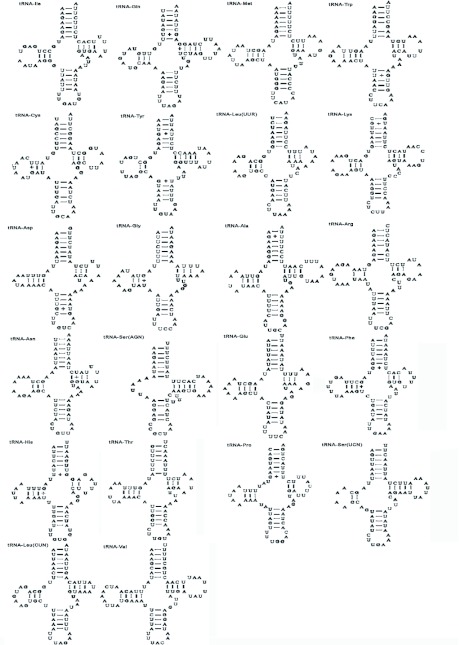
Inferred secondary structure of 22 tRNAs of *Sivaloka damnosus*. The tRNAs are labeled with the abbreviations of their corresponding amino acids. Nucleotide sequences are from 5′ to 3′ as indicated for tRNAIle. Watson-Crick base pairs designated by “-” and G-U base pairs by “+”. High quality figures are available online.

The standard 22 tRNA genes were identified in the same relative genomic positions as observed for the *Drosophila yakuba* genome (Clary and Wolstenholme 1984). The predicted secondary structure of 22 tRNA genes in the *S. damnosus* mitogenome is shown in Figure 2. All tRNAs have the typical clover-leaf structure except for tRNA*^Ser(AGN)^*, which has a reduced DHU arm; this is also the case in several metazoan mtDNAs, including insects (Wolstenholme 1992). The sizes of tRNA genes in *S. damnosus* range from 59 to 73 bp. All tRNA genes possess invariable length of 7 bp for the aminoacyl stem, 7 bp for the anticodon loop, and 5 bp for the anticodon stem. Therefore, most of the size variability in the tRNAs originates from length variation in the DHU arms and T ψ C arms. A total of 26 unmatched base pairs are scattered in fifteen tRNA genes of *S. damnosus*. Seventeen of them are G-U pairs, which form a weak bond. The remaining are A-A, G-A and U-U mismatches.

**Figure 3.**
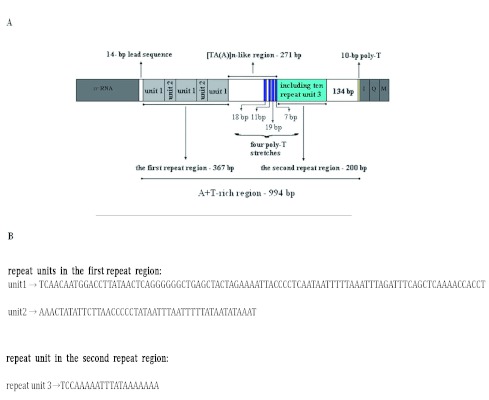
(A) The structural organization of the A+T-rich region of *Sivaloka damnosus*. The A+T-rich region flanking genes *srRNA*, tRNA^Ile^ (I), tRNA^Gln^ (Q), and tRNA^Met^ (M) are represented in grey boxes. (B) The sequences of the repeat units in the first repeat region and the second repeat region. High quality figures are available online.

As in all other sequenced mitogenomes, two genes of rRNAs are present in *S. damnosus*. The boundaries of rRNA genes were determined by sequence alignment with those of *T. dimidiata* and *P. spumarius*. The large and small ribosomal RNA genes are 1,192 and 711 bp in length, respectively, with an A + T content of 78.5% and 76.8%, respectively.

Their lengths are shorter than those of *P. spumarius* (1,245 bp for *lrRNA*, and 754 bp for *srRNA*) and *T. dimidiata* (1,270 bp for *lrRNA*, and 781 bp for *srRNA*).

### A+T-rich region

The A+T-rich region is well known for the initiation of replication in both vertebrates and invertebrates, and the reduced G + C content is one of the most outstanding features of this region (Boore 1999). The 994-bp *S. damnosus* A+T-rich region is located in the conserved location between *srRNA* and tRNA*^Ile^* (Figure 3A), and has an A + T content of 81.7%. The A+T-rich region can be divided into five parts (Figure 3A) : (1) a 14-bp lead sequence following the small ribosomal gene, which is a [TA(A)]n-like sequence; (2) the 367-bp repeat reigon following the 14-bp lead sequence; (3) a [TA(A)]n-like region, which can be folded into several stem-and-loop structures, and contains four poly-T stretches at the 3' end on the minority strand; (4) the 200-bp tandem repeat region, which is composed of a 20-bp repeat unit (“TCCAAAAATTTATAAAAAAA” on the majority strand); (5) a 10-bp poly-T stretch near tRNA*^Ile^* gene on the majority strand.

### Phylogenetic relationships

Phylogenetic analyses based on the two data sets (DNA alignment of all three codon positions from the concatenated 13 protein-coding genes, and DNA alignment including only the 1st and 2nd codon positions) generated two similar topologies. Both results supported the hypothesis of (Heteroptera+(Cicadomorpha+(Fulgoromorph a+Sternorrhyncha))) (Figure 5A). A monophyletic Sternorrhyncha and a monophyletic Pentatomomorpha were well recovered. Compared with the topology based on only first and second codon positions, in the tree based on all codon positions, Acyrthosiphon pisum+Schizaphis graminum was placed to the bottom of Sternorrhyncha and the inferred phylogenetic relationships of Lygaeoidea (Phaenacantha marcida+(Geocoris pallidipennis+ (Yemmalysus parallelus+Malcus inconspicuus))) were supported.

**Figure 4.**
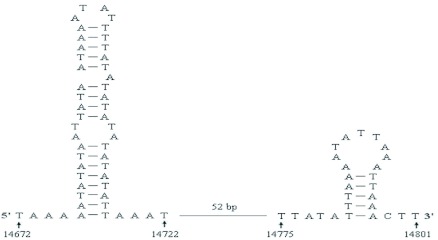
The potential secondary structures in the A+T-rich region of *Sivaloka damnosus*. High quality figures are available online.

In the analysis of rRNAs, Sternorrhyncha was found to be a sister group to all other Hemiptera. Similar to the analysis with nucleotide sequences of the protein-coding genes, a monophyletic Fulgoromorpha was well recovered based on two rRNA genes. However, *Homalodisca coagulate* was placed to be a sister position to the clade (*Geisha distinctissima* + *Sivaloka damnosus*), resulting in failure for the recovery of a monophyletic Cicadomorpha (Figure 5B). The low support value (BPP = 0.52) suggested that the position of *H. coagulate* was not well resolved in the phylogenetic analysis. In addition, the monophyletic Lygaeoidea were not recovered.

**Figure 5A.**
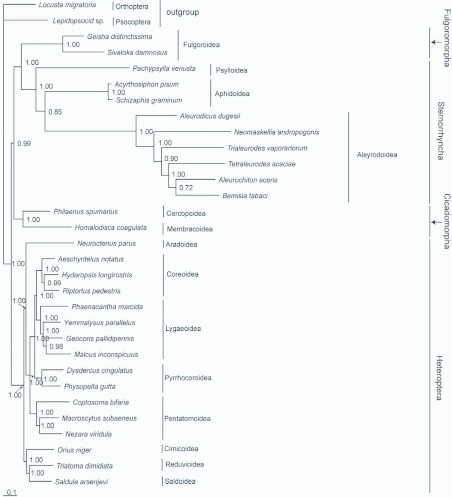
Phylogenetic analyses were based on first and second codon positions of the concatenated 13 protein-coding genes (A) and the concatenated 2 rRNA genes (B). The trees were rooted by Psocoptera and Orthoptera. Numbers refer to Bayesian posterior probabilities (BPP; near nodes). High quality figures are available online.

## Discussion

The size (15,287 bp) of the complete *S. damnosus* mitogenome is well within the observed range of insect mitogenomes (14–19 kb). The orientation and gene order are identical to the hypothesized ancestral arthropod arrangement found in several insect orders such as Diptera (Lewis et al., 1995).

The A + T content (76.5%) of the *S. damnosus* mitogenome is very close to the mean observed for other insects. Average A + T content of tRNA (77.1%) and rRNA genes (77.9%)) are higher than that of protein-coding genes (75.7%) (Table 4). In *S. damnosus*, the strongest A + T bias is found in the sites that evolve under low purifying selection pressure such as the A+T-rich region (81.7%) or the third codon positions (majority strand: 82.6%,minority strand: 86.2%). On the other hand, the A +T content of the first codon positions (majority strand: 72.7%, minority strand: 74.5%) and the second codon positions (majority strand: 66.3%%, minority strand: 74.1%)) are the lowest in the mitogenome of *S. damnosus*. These data suggest that the A + T bias might be introduced by mutational pressure as has been found in other mitogenomes (Foster et al. 1997).

**Figure 5B.**
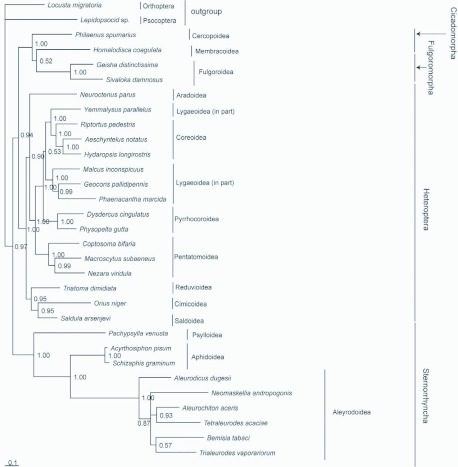
Phylogenetic analyses were based on first and second codon positions of the concatenated 13 protein-coding genes (A) and the concatenated 2 rRNA genes (B). The trees were rooted by Psocoptera and Orthoptera. Numbers refer to Bayesian posterior probabilities (BPP; near nodes). High quality figures are available online.

Interestingly, despite the overall lower A + T content, the second codon positions have a higher content of T than the A+T-rich region. G is underrepresented compared to C in the third codon positions of protein-coding genes in the majority strand, which is in line with the general trend towards lower G content in the mitogenome (Lessinger et al. 2000). However, in the *S. damnosus* mitogenome, the opposite bias is observed in the third codon positions of protein-coding genes in the minority strand. Although the exact reason for strand asymmetry in mtDNA is unknown, one possible reason is the accumulation of mutations in different strands, caused by strands being displaced during the replication cycle (Reyes 1998).

In the mitogenome of *S. damnosus*, six of the protein-coding genes, *nad2, cox1, cox2, nad3, nad5*, and *nad1*, are flanked by tRNA genes on both the 5′- and 3′-ends. Among the remaining seven protein-coding genes, four are adjacent to another protein-coding gene at their 3'-end region, *atp8, atp6, nad4l*, and *nad6*, which are arranged as *atp8-atp6, atp6-cox3, nad4l-nad4*, and *nad6-cytb*. These are three sets of overlapping genes, *atp8-atp6, nad6-cytb*, and *nad4l-nad4*, and one set of abutting genes, *atp6* with *cox3*. It has been proposed that the secondary structure of the transcribed polycistronic mRNA may facilitate cleavage between the proteins (Clary and Wolstenholme 1985). Potential secondary structures forming are present at the 3′-end of the upstream protein-coding genes, *atp6*, and *nad6*, which may act as signals for the cleavage of the polycistronic primary transcript (Clary and Wolstenholme 1985).

The average A + T content of the tRNAs was 77.1%), thus higher than that of the genome as a whole. The anticodons of the *S. damnosus* tRNAs are identical to those in *D. yakuba* (Clary and Wolstenholme 1985), *T. dimidiata* (Dotson and Beard 2001) and *P. spumarius* (Stewart and Beckenbach 2005). A total of 26 unmatched base pairs have been observed in the stems of *S. damnosus* tRNA secondary structures. This number is similar to other hemipteran species (e.g., *P. spumarius*). Yokobori and Pääbo (1995) demonstrated that in some metazoan mitochondrial tRNA genes, such mismatches can be corrected by RNA editing. Thus, the mismatches in the genomic aminoacyl stem sequences may not cause any difficulty in the transportation of the respective amino acids.

The presence of poly-T Stretchs have been reported in the A+T-rich region of other insects (Zhang et al. 1995; Saito et al. 2005). Similarly, in *S. damnosus*, there is a 10-bp poly-T stretch near tRNA*^Ile^* gene on the majority strand, whose location is the same with a 16–21 bp poly-T stretch in *Drosophila* (Saito et al. 2005). It has been speculated that this poly-T stretch may be involved in transcriptional control or may be the site for initiation of replication (Clary and Wolstenholme 1987; Lewis et al. 1994; Zhang et al. 1995). Besides this poly-T stretch, four poly-T stretches at the 3'-end of the [TA(A)]n-like region exist on the minority strand (Figure 3A). Their function is unknown but may be similar to the 10-bp poly-T stretch. Further studies are needed for comparing these sequences to see if they are functional.

The stem-and-loop structure in the A+T-rich region is believed to play a signaling role in mitogenome replication (Zhang and Hewitt 1997). In contrast to the primary sequence divergence in the secondary structure, the sequence flanking the structure is highly conserved among several insect orders, having consensus sequences of “TATA” at the 5′ end and “G (A) nT” at the 3'-end (Zhang et al. 1995; Schultheis et al. 2002). However, we found “CTT” rather than “G (A) nT” at the 3' end in the *S. damnosus* A+T-rich region (Figure 4). The presence of such structures flanked by conserved sequences in diverse insect orders was suggested as the site of the second strand replication origin (Zhang et al. 1995). However, the A+T-rich region of some insects, for example, *Coreana raphaelis* and *P. spumarius* (Kim et al. 2006; Stewart and Beckenbach 2006), exhibiting the potential to form stable secondary structures, did not show conservation of flanking motifs. Thus, it seems that the immediately flanking sequence may exist as a different form, or such a sequence may not be universally conserved in insects.

Immediately after the 14-bp lead sequence, is the first repeat region in the A+T-rich region of *S. damnosus*. This region is composed of two sets of complicated and long-winded repeat units (Figure 3B). And these repetitive sequences are arranged alternately, in which 92-bp unit 1 repeats three times and 46-bp unit 2 repeats two times. The arrangement of the two sets of repeat units is shown in Figure 3A. The A+T-rich region of *S. damnosus* also has a second repeat sequence 200 bp in length including a 20-bp repeat unit tandemly repeated ten times. In this repeat sequence, the ninth repeat unit has a single T to C transversion at position 13, and the tenth repeat has two transversions of A to C at position 17 and 18. The presence of two sets of repetitive regions has previously been found in the mitogenome A+T-rich regions of *Drosophila. melanogaster* (Lewis et al. 1994) and *P. spumarius* (Stewart and Beckenbach 2006). In *S. damnosus*, two sets of repeat regions are separated by nonrepetitive sequences, which is similar to *D. melanogaster* and *P. spumarius*. However, there is limited homology among the three species. Therefore, the resemblance among the repeat structures in the three species is superficial and non-homologous.

Although the obvious regularity of these repeat elements makes them of keen interest, the reason for the occurrence is unknown. Repeat sequences composed of tandem units are common in the metazoan A+T-rich region, and the length variation of A+T-rich region is predominantly due to variable numbers of repeat unit copies (Broughton and Dowling 1994; Wilkinson et al. 1997). Analyses of length variation of tandem arrays among numerous species have not been found informative in resolving geographical structuring or phylogenetic relationships (Broughton and Dowling 1994; Wilkinson et al. 1997). In crickets, however, the tandem repeat sequences appear to have undergone concerted evolution, and the nucleotide sequences of repeat units appear to be homogeneous within an individual or population but are heterogeneous among repeat units of divergent populations or species (Rand 1994). Thus, more data will be necessary for a decisive conclusion.

The ordinal classification and evolutionary affiliations of higher taxa in Hemiptera have been debated from the time Linneaus originally established this order in 1758. Using morphological data, Hamilton (1981) rasised a phylogenetic hypothesis of ((Heteroptera+Coleorhyncha)+(Fulgoromorph a+(Cicadomorpha+Sternorrhyncha))).

However, some molecular methods based on the partial 18S rDNA nucleotide sequences (Wheeler et al. 1993; von Dohlen and Moran 1995; Campbell et al. 1995) support the hypothesis that Sternorrhyncha is a sister-clade to all other Hemiptera. Our phylogenetic analyses based on the mitochondrial protein-coding gene sequences support this hypothesis, and further suggest that Cicadomorpha is the sister group of clade Fulgoromorpha+Sternorrhyncha. The genealogical proximities of hemipteran lineages are similar to Hamilton (1981). The differences between the topologies based on all codon positions and only first and second codon postions show that substitution saturation at the third codon position of protein-coding genes have an effect on the phylogenetic analysis. Here, we regard that the analysis excluding the third codon position is more rational. The phylogenetic analysis of Pentatomomorpha based on first and second codon positions is the same as Hua et al (2008), whose study was based on all 37 genes of mitogenome.

The analysis using two rRNA genes leads to quite different tree topologies, compared to the tree based on protein-coding genes. Sternorrhyncha is placed in a sister position to all other Hemiptera, and (Cercopoidea+ (Membracoidea+Fulgoroidea)) is the sister group of Heteroptera. This result is similar to Campbell et al (1995). In the topology based on rRNAs, the monophyletic Cicadomorpha and the monophyletic Lygaeoidea were not proved, and some nodal support values were relatively low (0.52 for Membracoidea+Fulgoroidea, and 0.53 for Lygaeoidea (in part)+(Coreoidea+Lygaeoidea (in part))). This suggests that the mitochondrial rRNA genes may not be suitable to estimate phylogenetic relationships of higher taxa in Hemiptera.

Different results inferred from different gene types indicate that more species and data are required to resolve the phylogenetic relationships within Hemiptera. Further studies on hemipteran species' mitogenome sequences should be instructive.
